# *Lactobacillus brevis* 23017 Relieves Mercury Toxicity in the Colon by Modulation of Oxidative Stress and Inflammation Through the Interplay of MAPK and NF-κB Signaling Cascades

**DOI:** 10.3389/fmicb.2018.02425

**Published:** 2018-10-12

**Authors:** Xinpeng Jiang, Shanshan Gu, Di Liu, Lili Zhao, Shuang Xia, Xinmiao He, Hongyan Chen, Junwei Ge

**Affiliations:** ^1^Heilongjiang Key Laboratory for Animal Disease Control and Pharmaceutical Development, College of Veterinary Medicine, Northeast Agricultural University, Harbin, China; ^2^Key Laboratory of Combining Farming and Animal Husbandry, Ministry of Agriculture, Heilongjiang Academy of Agricultural Sciences, Animal Husbandry Research Institute, Harbin, China; ^3^Heilongjiang Provincial Key Laboratory of Laboratory Animal and Comparative Medicine, State Key Laboratory of Veterinary Biotechnology, Chinese Academy of Agricultural Sciences, Harbin Veterinary Research Institute, Harbin, China

**Keywords:** *Lactobacillus brevis*, mercury, oxidative stress, inflammation, MAPK, NF-κB

## Abstract

**Aims:** *Lactobacillus* strains have protective effects against heavy metals while relieving oxidative stress and modulating the immune response. Mechanisms that ameliorate heavy metal toxicity and the relationship between probiotics and gut barrier protection in the process of heavy metal pathogenesis was poorly understood.

**Methods and Results:** In this study, *Lactobacillus brevis* 23017 (LAB, *L. brevis* 23017), a selected probiotics strain with strong mercury binding capacities, was applied to evaluate the efficiency against mercury toxicity in a mouse model. Histopathological results along with HE stains show that *L. brevis* 23017 protects the integrity of the small intestinal villus, which slows weight loss in response to Hg exposure. The qRT-PCR results demonstrate that *L. brevis* 23017 maintains a normal mucosal barrier via modulation of tight junction proteins. Importantly, the present study demonstrates that *L. brevis* 23017 effectively ameliorates injury of the small intestine by reducing intestinal inflammation and alleviating oxidative stress in animal models. Moreover, *L. brevis* 23017 blocks oxidative stress and inflammation through MAPK and NF-κB pathways, as shown by western blot.

**Conclusions:** Together, these results reveal that *L. brevis* 23017 may have applications in the prevention and treatment of oral Hg exposure with fermented functional foods by protecting gut health in daily life.

## Introduction

Mercury (Hg) is one of the most toxic elements, existing in a variety of sources in the environment, including fish and plants (Elisavet et al., 2014; Perello et al., 2015); it is introduced into the environment by industries that use electroplating, paint, and mining (Cano-Sancho et al., 2015). Hg exposure to different human organs adversely affects human health, contributing to renal failure, bone structure deformity, reproductive disease, cardiovascular dysfunction, nervous system dysfunction, inflammation and several cancers (Sakamoto et al., 2015, 2017). Moreover, recent research has revealed that oral Hg exposure affects the gut ecology, increasing inflammation and susceptibility to colitis in mice (Stejskal, 2013). Research has shown that Hg exposure causes serious intestinal disease, including damage to tight junction proteins and necrosis of epithelial cells through an inflammatory response (Toomey et al., 2014; Eaton et al., 2017). Disruption of the intestinal barrier has been related to gut immunity and oxidative stress induced by Hg exposure (Laporte et al., 2002; Hoyle and Handy, 2005).

Several studies have revealed that specific *Lactobacillus* strains alleviated heavy metal toxicity (Tian et al., 2015; Trinder et al., 2016; Yu et al., 2016; Alcantara et al., 2017). Recently, Majlesi M et al have proved the *L. plantarum* and *Bacillus coagulans* protected significantly against mercury toxicity in liver and kidney in rats by decreasing the mercury, creatinine, urea, bilirubin, ALT, and AST level, and preventing alterations in the levels of GPx and SOD (Majlesi et al., 2017). And there were a lot of research on the lactobacillus attenuates inflammation in mice by inhibiting NF-κB signaling pathway (Lim et al., 2017). Both of the MAPK and NF-κB have been widely accepted to suppress the oxidative stress and inflammation by specific *Lactobacillus* via inhibiting NF-κB and p38 MAPK pathways (Chon et al., 2010; Saito et al., 2012). However, more studies on the protective effects of *Lactobacillus* against Hg toxicity, and corresponding protective mechanisms need to be examined. The intestinal epithelium, the largest barrier between the body and the environment, processes and transports many nutrients and does not have extensive interaction with the environment. Therefore, the small intestine is one of the first barriers to potentially harmful factors that may enter the body. From the perspective of intestinal barrier function, it is possible to find some new discoveries.

Our previous study demonstrated that *Lactobacillus brevis* 23017 possess strong Hg and heavy metal binding capacities (Jintai et al., 2017). Here, the present study aimed to analyze the effect of *Lactobacillus brevis* 23017 on Hg toxicity by preservation of intestinal health in a mouse model. We hypothesized that *Lactobacillus* would absorb Hg in the gut of mice and that the decrease of Hg would relieve the lesions that compromise intestinal anti oxidative stress and modulate the inflammatory response. Our research aim to find that the regulatory mechanism of *Lactobacillus* anti-oxidative stress and anti-inflammation in this study involved the P38 and NF-κB pathways in the different intestinal segment of duodenum and colon.

## Materials and methods

### Animal experiments

The Ethical Committee of the Institute approved all scientific experiments. All applicable international and national guidelines for the care and use of animals in experiments were followed. Approval (2016NEAU-219, 13 September 2016) was obtained from the Institutional Committee of Northeast Agricultural University for animal experiments. Mercury chloride (HgCl_2_, MW = 183.03, analytical grade) was purchased from Sigma-Aldrich (MO, USA). Mercury solutions with varying concentrations of HgCl_2_ were prepared in distilled water. Five groups of 4-week-old female BALB/c mice (Beijing Vital River Laboratory Animal Technology Co., Ltd) were used in the experiments, with each group containing 10 mice. Animal procedures for 5 groups are shown in Table 1. (1) *L. brevis* 23017 was suspended in MRS medium, and MRS alone served as the control group. The control group (*n* = 10 per group) was orally dosed with 200 μL MRS medium during the experimental period. (2) The Hg-5d group was dosed with 200 μL MRS medium for the first 5 days, and on the sixth day this group was given oral solutions of Hg at a final concentration of 10 mg/kg (1/5 LD50). (3) The LAB+ Hg-5d group was dosed with *L. brevis* 23017 (10^9^ CFU/200 μL) for the first 5 days, and on the sixth day this group was given oral solutions of Hg (10 mg/kg). (4) The Hg-12d group was dosed with 200 μL MRS media for the first 5 days, this group was given oral solutions of Hg at a final concentration of 10 mg/kg on the 6th day, and this group continued to be dosed with MRS media for the last 6 days. (5) The LAB+ Hg-12d group was dosed with *L. brevis* 23017 (10^9^ CFU/200 μL) for the first 5 days, was given oral solutions of Hg at a final concentration of 10 mg/kg on the 6th day, and this group continued to be dosed with *L. brevis* 23017 (10^9^ CFU/200 μL) for the last 6 days (Brandao et al., 2006). All mice had *ad libitum* access to standard rodent chow and distilled water throughout the study. All functional measurements were carried out 48 h after the last toxin administration, and 12 h following no oral intake. Tissues were harvested from animals anesthetized by injection of 40 mg/kg b.w. of sodium thiopental. Body weight of the mice was measured throughout the experiment. During the experimental period, each mouse was moved into a clean, empty cage to obtain body weight. The Hg-5d and LAB+ Hg-5d groups were tested for 7 days, and the Hg-12d and LAB+ Hg-12d groups were tested for 12 days.

**Table 1:** Animal experimental protocol.

**Group**	**Treatment time**					
	**1–5 d**	**6 d**	**7 d**	**8 d**	**9–12 d**	**13 d**
Control	MRS	Water	Observation	Sample	MRS	Sample
LAB+Hg-5d	LAB	Hg	Observation	Sample	–	–
Hg-5d	MRS	Hg	Observation	Sample	–	–
LAB+Hg-12d	LAB	Hg	LAB	LAB	LAB	Sample
Hg-12d	MRS	Hg	MRS	MRS	MRS	Sample

*The MRS was the lactobacillus medium, which as the normal ways. The LAB was the L. brevis 23017, which was resuspended with MRS. The Hg means the Hg expose, which was dissolved with water. Observation means that there was no experiment except the observation in this group. The sample means the sample collection*.

### Mercury determination

Mercury content in the feces and muscle was determined by an atomic absorption spectrometry graphite tube technique (AAS Varian 1275; graphite tube, GTA-95, Palo Alto, Ca, USA). In brief, pieces of tissue were dried for 4 h at 60°C followed by the addition of 65% HNO_3_ to the dried tissue and digestion with a high-performance microwave system. A solution prepared from the digested sample and Milli-Q water was used for determination of Hg levels by graphite furnace atomic absorption spectrometry. Reference materials were used as controls. All dilutions were performed using metal-free, ultrapure water. Solutions were stored in 25 ml high-density polyethylene vials at 4°C prior to analysis. Concentrations are expressed as mg of Hg per kg of feces and muscle (Sun et al., 2014).

### Gross lesion and histopathological examinations

Intestinal tissues were collected from standardized areas of the duodenum, jejunum, ileum and colon within 10 to 15 min post mortem. Intestinal and other major organ tissue samples were fixed and stained according to routine histological methods (Trajkovic et al., 2007). After immediate fixation in 10% buffered formalin, samples were embedded in paraffin wax. Paraffin sections (4 μm) were stained with hematoxylin–eosin (H&E) for routine histological examination, and sample histopathology was examined using a light microscopy (Olympus, Japan).

### Antioxidant activity assay

Antioxidant assays on intestinal tissues with Lactobacillus were performed by measuring the following: GSH, SOD, CAT and MDA. Briefly, 0.5 g of intestinal tissue was homogenized in 1 ml PBS using a homogenizer and centrifuged at 12,000 rpm (13,400 g) for 10 min. Supernatants were collected and stored at −80°C for analysis. Intestinal antioxidant activity was determined using a GSH assay kit, SOD assay, CAT assay kit and MDA assay kit, all of which were purchased from Nanjing Jiancheng Bioengineering Institute. Activity of SOD was determined by the epinephrine method (Yokoyama et al., 1993). SOD activity was assayed using the xanthine/xanthine oxidase method based on the production of O^2−^ anions at pH 10.2. CAT activity was determined by the rate of H_2_O_2_ decomposition measured spectrophotometrically at 240 nm, as previously described (Zhang et al., 2014). GSH activity was estimated based on its catalysis by the oxidation of reduced glutathione in the presence of cumene hydroperoxide. Generation of nicotinamide adenine dinucleotide phosphate was measured spectrophotometrically at 340 nm. Lipid peroxidation was evaluated by measuring MDA concentrations according to thiobarbituric acid (Mao et al., 2012). MDA concentrations were calculated by the absorbance of TBA reactive substances at 532 nm. Activities of SOD and CAT are expressed as units per milligrams of protein (U/mg protein). Activity of GSH and MDA are expressed as μmol/g protein and nmol/mg protein, respectively.

### Real-time quantitative RT-PCR (qRT-PCR) analysis

We employed real-time qRT-PCR to determine the levels of cytokine gene products in intestinal samples using a CFX96TM Real-Time PCR Detection System (Bio-Rad). Total RNA was extracted from intestinal tissues using total RNA extraction kits (iNtRON) according to the manufacturer's instructions. Purified RNA was then converted to cDNA using EasyScript First-Strand cDNA Synthesis SuperMix (TransGen Biotech, Beijing, China). The resulting cDNAs were used for real-time PCR with SYBR® qRT-PCR reagent kit (Thermo Scientific, PA, USA), and the specific primers used are listed in Table 2 (Cheng et al., 2014). The Livak method (2^−ΔΔ*CT*^ method) was used to calculate the fold change compared to β-actin gene controls.

**Table 2:** Primers used for q-RT PCR for inflammatory and functional analyses.

**Primer**	**Forward sequence**	**Reverse sequence**
β-actin	CCAGTTGGTAACAATGCCATGT	GGCTGTATTCCCCTCCATCG
IL-6	AAAGAGTTGTGCAATGGCAATTCT	AAGTGCATCATCGTTGTTCATACA
IL-10	CGCAGCTCTAGGAGCATGTG	GCTCTTACTGACTGGCATGAG
TNF-α	AGTGGTGCCAGCCGATGGGTTGT	GCTGAGTTGGTCCCCCTTCTCCAG
IL-4	ATGGGTCTCAACCCCCAGCTAGT-	GCTCTTTAGGCTTTCCAGGAAGTC-
occludin	GCTGTGATGTGTGTGAGCTG	GACGGTCTACCTGGAGGAAC
ZO-1	AGGACACCAAAGCATGTGAG	GGCATTCCTGCTGGTTACA

### Western blot analysis of P38 and NF-κB

Intestinal segments were washed with cold PBS and then lysed with RIPA Lysis and Extraction Buffer (Thermo Fisher, USA). Protein concentrations were determined by BCA protein assay kit (Pierce Chemical Co.). One hundred microgram of protein extracts were loaded and electrophoresed on 12% SDS polyacrylamide gels and transferred to PVDF membranes (Bio-Rad, USA). The following primary antibodies were utilized: anti-P38, anti-p-P38, anti-NF-κB, anti-p-NF-κBand anti-β-actin (Cell Signaling Technology, Inc., USA). The secondary antibody used for detection was conjugated to horseradish peroxidase. Enhanced chemiluminescence (ECL) was used to detect the conjugated horseradish peroxidase. The relative expression of specific bands was analyzed and quantified using ImageJ Software (NIH, Bethesda, MD, USA).

### Data analysis

All data shown in figures and tables are expressed as the mean ± SEM. One-way ANOVA (SPSS 15.0) was used for data comparisons between multiple groups. In each case, *P* < 0.05 was considered statistically significant.

## Results

### Body weight of mice

**Figure 1:**
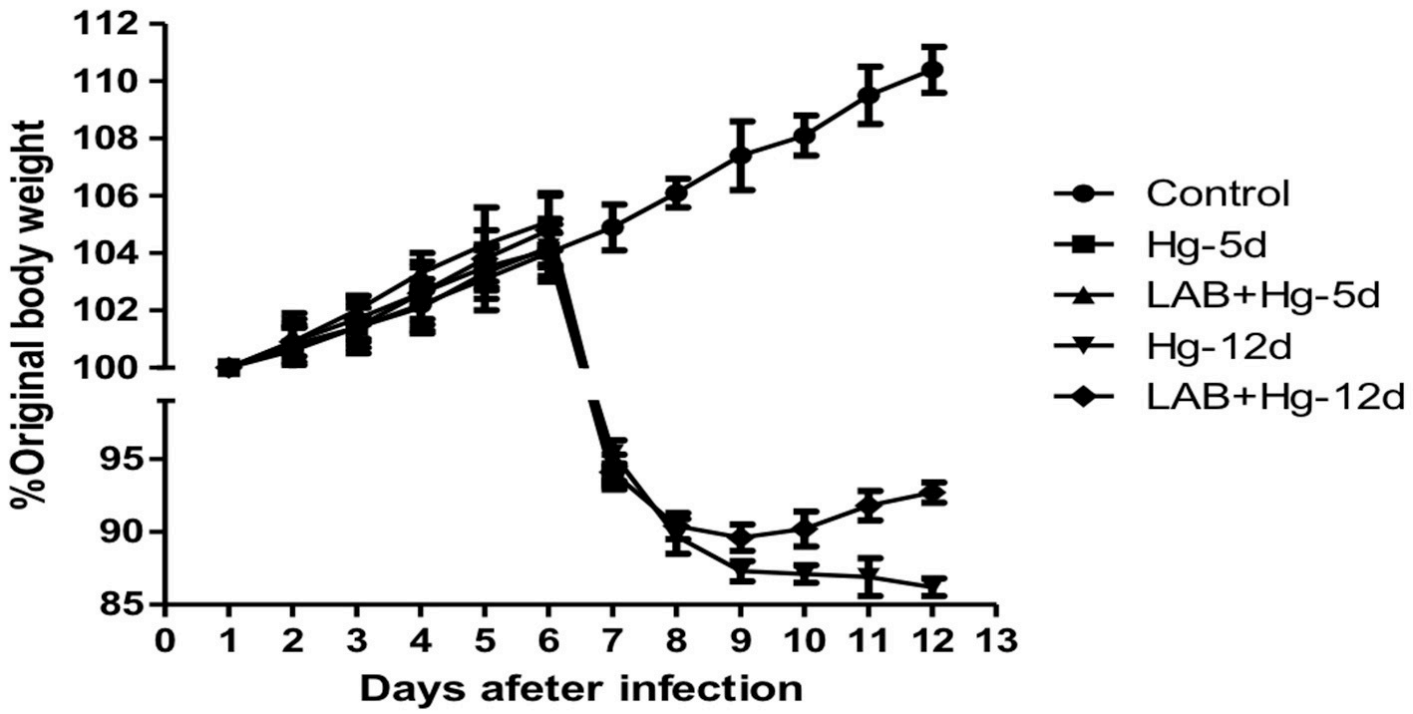
Changes in body weight observed among the five groups of animal experiments. Original body weight is designated as 100%. After expose to mercury chloride, results show that body weight in all groups was decreased relative to control.

Changes in body weight were observed among all five groups of mice, as shown in Figure 1. Original body weight was considered 100%. After expose to mercury chloride, body weight in all groups decreased, with the exception of controls, whose body weight steadily increased. Body weight in both the LAB+Hg-5d and Hg-5d groups decreased by 10.04 and 8.91%, respectively, within 48 h. These data reveal that while exposure to mercury chloride decreased body weight, the oral *Lactobacillus* in the LAB+Hg-5d group decreased more slowly than did the Hg-5d group. The body weights of the LAB+Hg-12d and Hg-12d groups also decreased in response to mercury exposure, but the rate of decrease was not as steep on days 8 and 9 compared with other days. Furthermore, we observed the changes of body weights in the LAB+Hg-12d and Hg-12d groups exhibited a point of inflection on the tenth day of exposure to mercury chloride, with the body weight of mice in the LAB+Hg-12d group increasing on the eleventh day.

### Gross lesion and histopathological examinations

**Figure 2:**
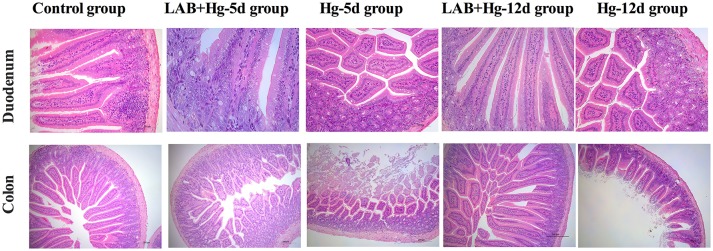
Microscopic examination revealed obvious differences in the five groups in small intestine samples from duodenum and colon. Histopathological examination of H&E-stained small intestinal tissues were analyzed in control, LAB+Hg-5d, Hg-5d, LAB+Hg-12d and Hg-12d groups. The top pictures illustrate pathological changes in the jejunum, and the scale bar is 100 μm. The bottom pictures show pathological changes in the ileum, and the scale bar is 200 μm. Histological pictures are representative of sections derived from each group.

Microscopic examination revealed obvious differences among the five groups of mice with respect to their small intestine samples taken from different intestinal segments. As indicated in Figure 2, there were no significant gross or microscopic lesions noted in the control group. In the LAB+Hg-5d group, however, there was inflammation and infiltration in the stratum propria, which exhibited vacuolization in the bottom of the stratum propria of both the duodenum and colon. The sloughing off of degenerate, necrotic enterocytes along with vacuolization was apparent in the duodenum and colonic cells of the Hg-5d group. There were no significant gross or microscopic lesions in the LAB+Hg-12d group in the duodenum. However, the Hg-12d exhibited duodenum inflammation and infiltration in the stratum propria. These same findings were observed in the colon. Both Hg exposure groups, Hg-5d and Hg-12d, presented with ruptured small intestinal cells. The LAB groups, LAB+Hg-5d and LAB+Hg-12d, fared significantly better than did the Hg exposure groups. The LAB+Hg-5d and LAB+Hg-12d groups retained the relative integrity of the small intestinal villus, and the intestinal villus of the LAB+Hg-12d group was much longer than that in the LAB+Hg-5d group. Both the LAB+Hg-5d and LAB+Hg-12d groups exhibited retention of small intestinal villus integrity.

### Mercury concentration in fecal and muscle samples

**Figure 3:**
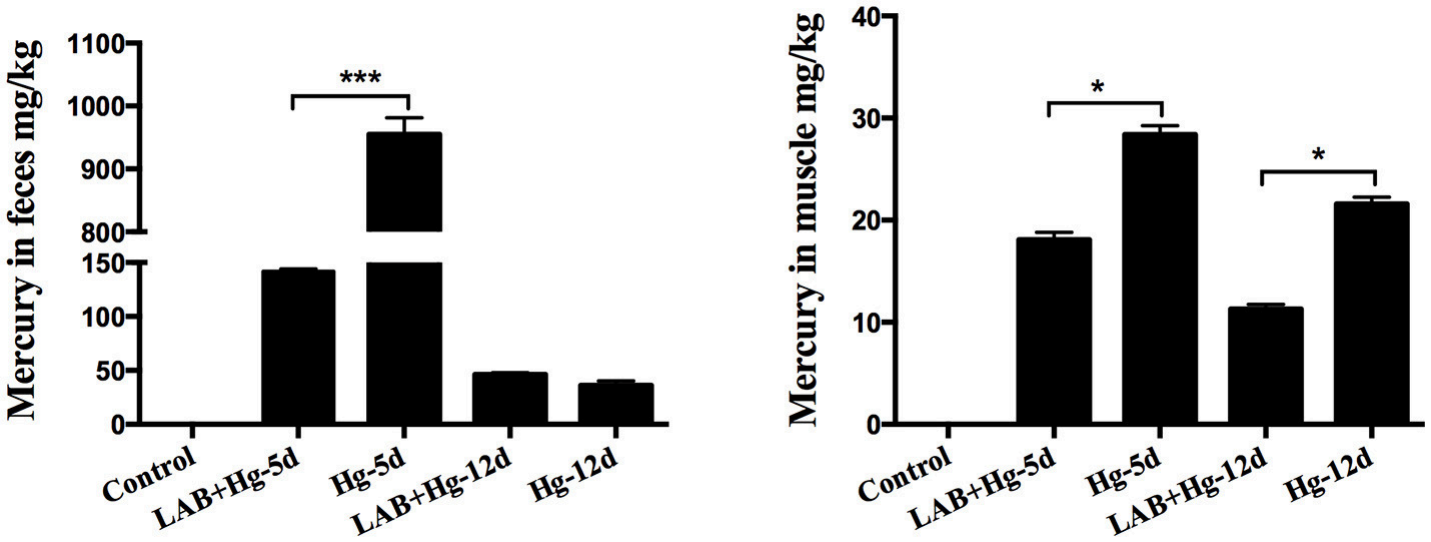
Analysis of Hg concentrations in tissue samples revealed dose-dependent increases in Hg levels. Hg was not found in the fecal samples of controls. Concentrations are expressed as mg of Hg per kg of feces and muscle. Asterisks indicate significant differences (*p* < 0.05). Results are represented as the mean ± SEM of three independent experiments.

Analysis of Hg concentrations in tissue samples and feces revealed dose-dependent increases in Hg levels. As expected, Hg was absent in fecal samples from the control group (Figure 3). The Hg concentrations of the Hg-5d and LAB+Hg-5d groups were ~955.03 and 141.87 mg/kg mg/kg in feces, respectively. Hg concentrations of the LAB+Hg-5d group were significantly lower than the Hg-5d group (*p* < 0.001). Fecal Hg concentrations of the LAB+Hg-12d group were ~36.21 mg/kg, which were lower than the Hg-12d group (46.28 mg/kg) in the feces. But there was no significant difference between these two groups. In this model of acute mercury poisoning, most of the mercury was discharged in the feces, but the discharged Hg was decreased in the 12-day groups. The feces results show that the Hg concentrations of LAB+Hg-12d and Hg-12d groups were lower than the LAB+Hg-5d and Hg-5d groups. A similar trend was observed for Hg concentrations in the muscle, but Hg concentrations were all significantly lower than in the feces. Most of the Hg was discharged in the feces, that is the reason the muscle absorbed lower Hg concentrations compared with feces. Furthermore, the Hg concentrations of the LAB+Hg-12d group (11.32 mg/kg) in muscle was also lower than the LAB+Hg-5d group (18.13 mg/kg). The Hg-5d group (28.41 mg/kg) also exhibited higher Hg concentration than did the Hg-12d group (21.65 mg/kg). Hg levels of the LAB+Hg-5d and LAB+Hg-12d groups in muscle were significantly higher than in the Hg-5d and Hg-12d groups. Both the LAB+Hg-5d group and LAB+Hg-12d group were significantly lower than the exposure groups(*p* < 0.05) in terms of Hg levels. Hg concentrations in the muscle highlight the importance of the LAB treatment in mitigating Hg accumulation in the body.

### Lab significantly inhibits antioxidant activity in Hg-exposed intestinal tissues

Activities of MDA, CAT, SOD and levels of GSH were used to evaluate antioxidant levels and are shown in Table 3. Hg exposure in Hg-5d and Hg-12d groups markedly increased levels of MDA, while decreasing SOD, CAT and GSH activities compared to the control group in both the duodenum and colon (*p* < 0.05). Compared to Hg-5d, LAB+Hg-5d significantly decreased activities of CAT and MDA, while increasing activities of SOD and GSH, a measure of oxidative stress, in the duodenum and colon (*p* < 0.05). Preventative assays illustrated that LAB increases CAT and SOD activities in the LAB+Hg-12d group compared to the Hg-12d group in the duodenum and colon (*p* < 0.05). LAB administration significantly decreased MDA levels in the LAB+Hg-12d group compared to the Hg-12d group in both duodenum and colon. Taken together, these data show that LAB effectively increases SOD and GSH activities and decreases CAT and MDA levels, indicators of oxidative stress. Duodenal SOD results demonstrate that the duodenum plays a more significant role in mediating oxidative stress than does the colon, as duodenal SOD activity was higher than colonic. GSH results suggest that the effects of mercury toxicity are primarily mediated by the duodenum. Oxidative damage in response to mercury toxicity was gradually reduced after the duodenum in the subsequent jejunum, ileum and colon.

**Table 3:** Antioxidant Indices in Duodenum and Jejunum Results.

**Intestinal segments**	**Groups**	**MDA (nmol/mgprot)**	**CAT (U/g Hb)**	**SOD (U/mgpro)**	**GSH (μmol/gprot)**
Duodenum	Control	1.77 ± 0.17	1.30 ± 0.12	8.57 ± 0.69	197.77 ± 2.04
	LAB+Hg-5d	5.51 ± 0.56^*^^#^	0.84 ± 0.26	7.60 ± 2.93^*^	105.56 ± 11.39
	Hg-5d	9.78 ± 2.52^#^	0.53 ± 0.21^#^	2.32 ± 1.34^#^	68.57 ± 8.29^#^
	LAB+Hg-12d	4.05 ± 0.72^*^^#^	0.91 ± 0.15^*^	7.03 ± 1.64^**^	112.10 ± 4.26^*^
	Hg-12d	7.68 ± 1.57	0.62 ± 0.26	1.28 ± 0.57^##^	79.72 ± 6.40^#^
Colon	Control	2.03 ± 0.46	1.34 ± 0.04	17.92 ± 2.84	54.55 ± 1.39
	LAB+Hg-5d	4.15 ± 0.31^*^	1.34 ± 0.16	17.61 ± 4.90^#^	48.11 ± 2.93^*^^#^
	Hg-5d	4.04 ± 0.21^*^	1.41 ± 0.13	16.44 ± 2.38^*^	44.61 ± 3.53^*^
	LAB+Hg-12d	3.36 ± 0.27^*^	1.30 ± 0.40	16.03 ± 1.95^*^	51.21 ± 1.45^#^
	Hg-12d	4.39 ± 0.30^*^^#^	1.27 ± 0.35	16.41 ± 1.55^*^	47.33 ± 2.41^*^

The activities of malondialdehyde (MDA), catalase (CAT), superoxide dismutase (SOD) and the level of glutathione(GSH) were used to evaluate antioxidant with intestinal tissues. Activities of SOD and CAT are expressed as units per milligrams of protein (U/mg protein). The activity of GSH and MDA are expressed as μmol/g protein and nmol/mg protein respectively. Data with ^*^ and ^#^ denote p < 0.05,^**^ and ^##^denote p < 0.01;

Data with ^*^ and ^**^represent each group comparing with the control group;

*Data with ^#^ and ^##^ represent LAB+Hg-5d group comparing with Hg-5d group or LAB+Hg-12d group comparing with Hg-12d. The results are represented as the mean ± SEM of three independent experiments*.

### Effects of lab on Hg-induced alteration of inflammation and intestinal integrity

**Figure 4:**
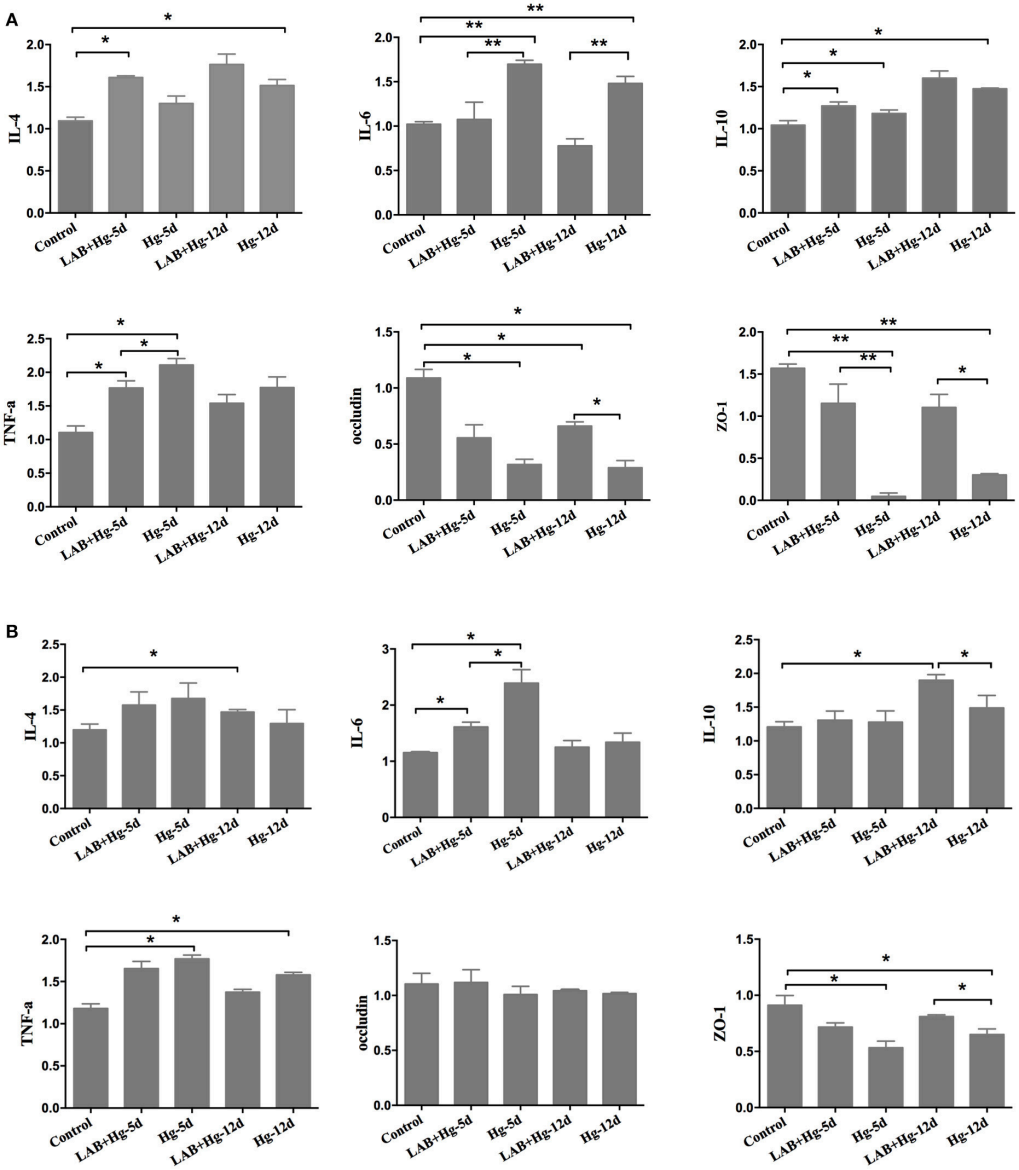
*Cytokines and intestinal integrity expression*. RNA expression was analyzed in both the duodenum and jejunum,including for TNF-α, IL-4, IL-6, IL-10, occluding, and ZO-1. **(A)** Gene expression of cytokines and integrity in the duodenum. **(B)** Results in the colon. The differences between means were considered significant at ^*^p < 0.05, very significant at ^**^p < 0.01. Results are represented as the mean ± SEM of three independent experiments.

Hg exposure significantly increased the expression of inflammatory cytokines, such as TNF-α, and IL-6, in both the duodenum (Figure 4A) and colon (Figure 4B). Additionally, Hg exposure significantly decreased intestinal integrity by decreasing the expression of ZO-1 in Hg-5d and Hg-12d groups compared to controls in the duodenum and colon. In the treatment, the LAB+Hg-5d group and LAB+Hg-12d group could effectively inhibit the inflammation in the duodenum and colon, such as the IL-6 and TNF-α. The inhibition of inflammation was not only the lactobacillus adsorbing Hg, but it could also block the inflammation itself. LAB also exerted anti-inflammatory properties, indicated by expression levels of IL-10 being higher in the LAB+Hg-5d and LAB+Hg-12d groups than in the control group in duodenum and colon. Addition of LAB in the LAB+Hg-5d/LAB+Hg-12d groups also significantly increased expression of ZO-1 compared to the Hg-5d/Hg-12d groups (*p* < 0.01) in the duodenum, indicating that LAB preserves duodenal function in response to Hg exposure. ZO-1 expression was also significantly greater in the LAB+Hg-12d group than in the Hg-12d group (*p* < 0.05). Occludin expression was significantly increased in the duodenum in the LAB+Hg-5d and LAB+Hg-12d groups compared with the Hg-5d and Hg-12d groups, and the occludin levels of Hg-5d and Hg-12d were lower than the control group (*p* < 0.05) in the duodenum, respectively. However, occludin expression was not significantly different in the colon between the different experimental groups. The mRNA expression of occludin showed that Hg did not affect intestinal integrity in the colon. The results of ZO-1 and occludin indicated that Hg had a different effect on intestinal integrity both in the duodenum and colon. The duodenum and colon have different biological functions.

### Lab inhibits oxidative stress and inflammation through the MAPK and NF-κB pathways

**Figure 5:**
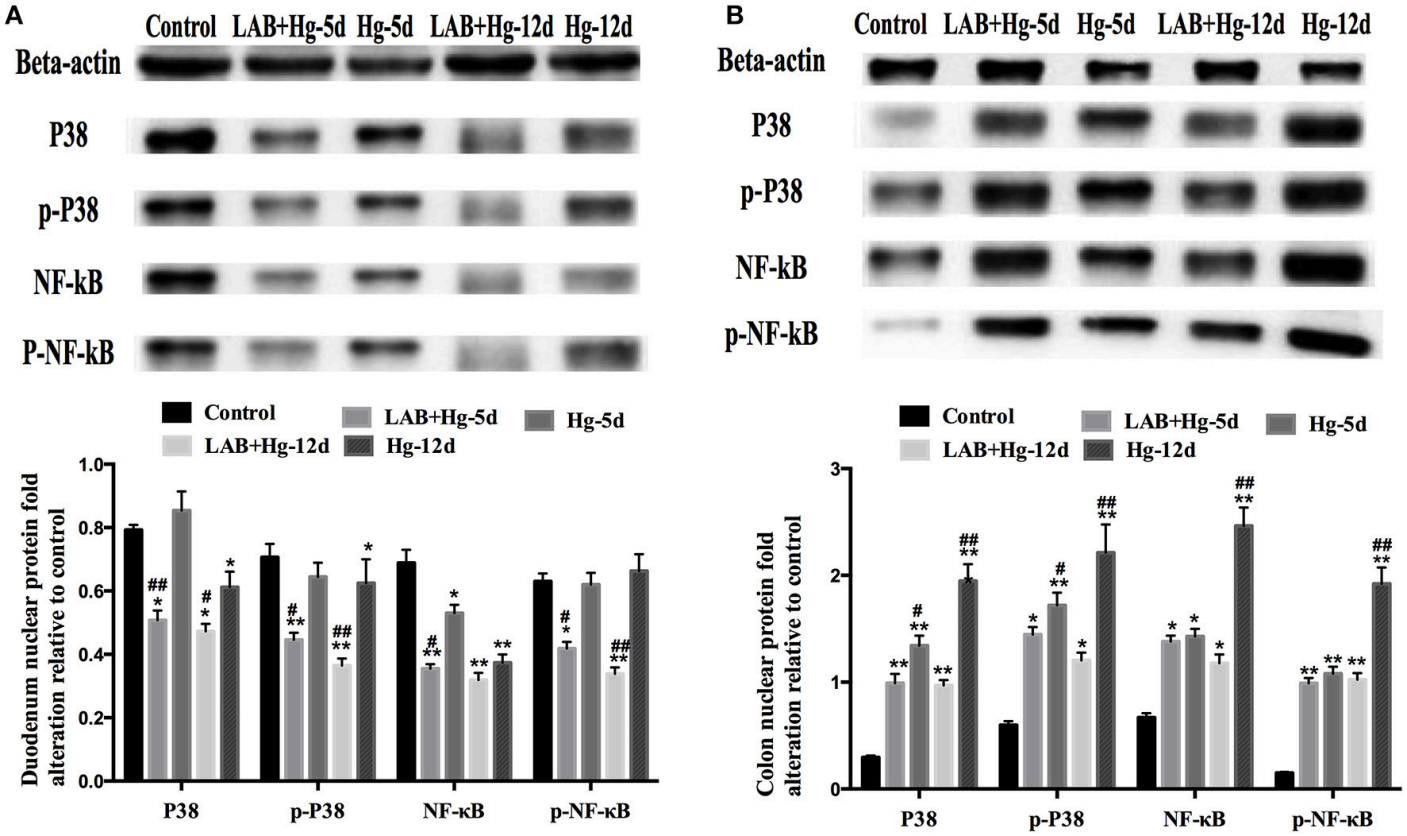
Potential role of MAPKs in inflammation observed after supplementation of LAB on Hg-exposed tissue as assessed by western blot. **(A)** Relative quantity duodenum P38, p-P38, *NF-*κ*B*, and p-*NF-*κ*B*. **(B)** Colonic results. ^*^and ^#^denote *p* < 0.05, ^**^and ^*##*^denote *p* < 0.01; Data with ^* and **^represent comparisons to the control group; ^#^ and ^*##*^represent LAB+Hg-5d group compared to Hg-5d group, or LAB+Hg-12d group compared to Hg-12d. Results are represented as the mean ± SEM of three independent experiments.

To determine the potential role of MAPKs in inflammation observed after LAB and Hg exposure, P38, *NF-*κ*B* and their phosphorylated forms were examined by western blot. As expected, Hg and LAB treatment significantly activated both P38 and *NF-*κ*B* proteins, indicating that both MAPKs and *NF-*κ*B* protein activation are induced during the acute stage of colonic lesion formation (Figure 5B). In contrast, Hg exposure did not induce either MAPKs or *NF-*κ*B* protein activation in the duodenum lesion (Figure 5A). After preventive LAB treatment, however, levels of phosphorylated P38 and *NF-*κ*B* and proteins were significantly reduced in the duodenum and colon. In the duodenum, the P38, p-P38 and *p-NF-*κ*B* proteins were inactivated in the LAB groups compared to the Hg groups. In colon, there were significant differences in phosphorylated P38 and *NF-*κ*B* proteins between Hg and LAB+Hg groups, wherein all protein levels were higher than in the control group, distinct from the duodenum. Hg and LAB treatment did not activate these proteins in the duodenum, whereas 5- and 12-day treatment with LAB were both effective in reducing expression of P38 and p-P38 in the colon. Compared to Hg-12d groups, treatment of LAB was only effective at reducing *NF-*κ*B* and *p-NF-*κ*B* expression in the LAB+Hg-12d groups in the colon. There were also significant reductions of P38 and phosphorylated proteins in the LAB+Hg-12d group compared to the Hg-12d group in the colon. In addition, LAB effectively reduced both P38 and p-P38 expression of lesions in the colon induced by Hg exposure. In the *NF-*κ*B* pathway, exposure of Hg plays a role in chronic injury and activation in the colon, and LAB exerted a long-term blockade of injury in the LAB+Hg-12d group. These results show that LAB primarily inhibits phosphorylation of P38 and *NF-*κ*B* in the duodenum, which was not the targeted intestinal segment. Exposure of Hg was mostly targeting colonic intestinal segments.

## Discussion

Our previous study confirmed the ability of a probiotic, *L. brevis* 23017, to inhibit Hg absorption in the intestines of mice, and the protective mechanism is partly due to intestinal excretion by the Hg-binding ability of the strain. Hg was absorbed by *L. brevis* 23017, leading to decreased Hg absorption in muscle and gut. This study was focused on evaluating whether probiotics can protect the gut barrier with other routes on the first targeting tissue of small intestine in addition to Hg absorption with *L. brevis* 23017.

**Figure 6:**
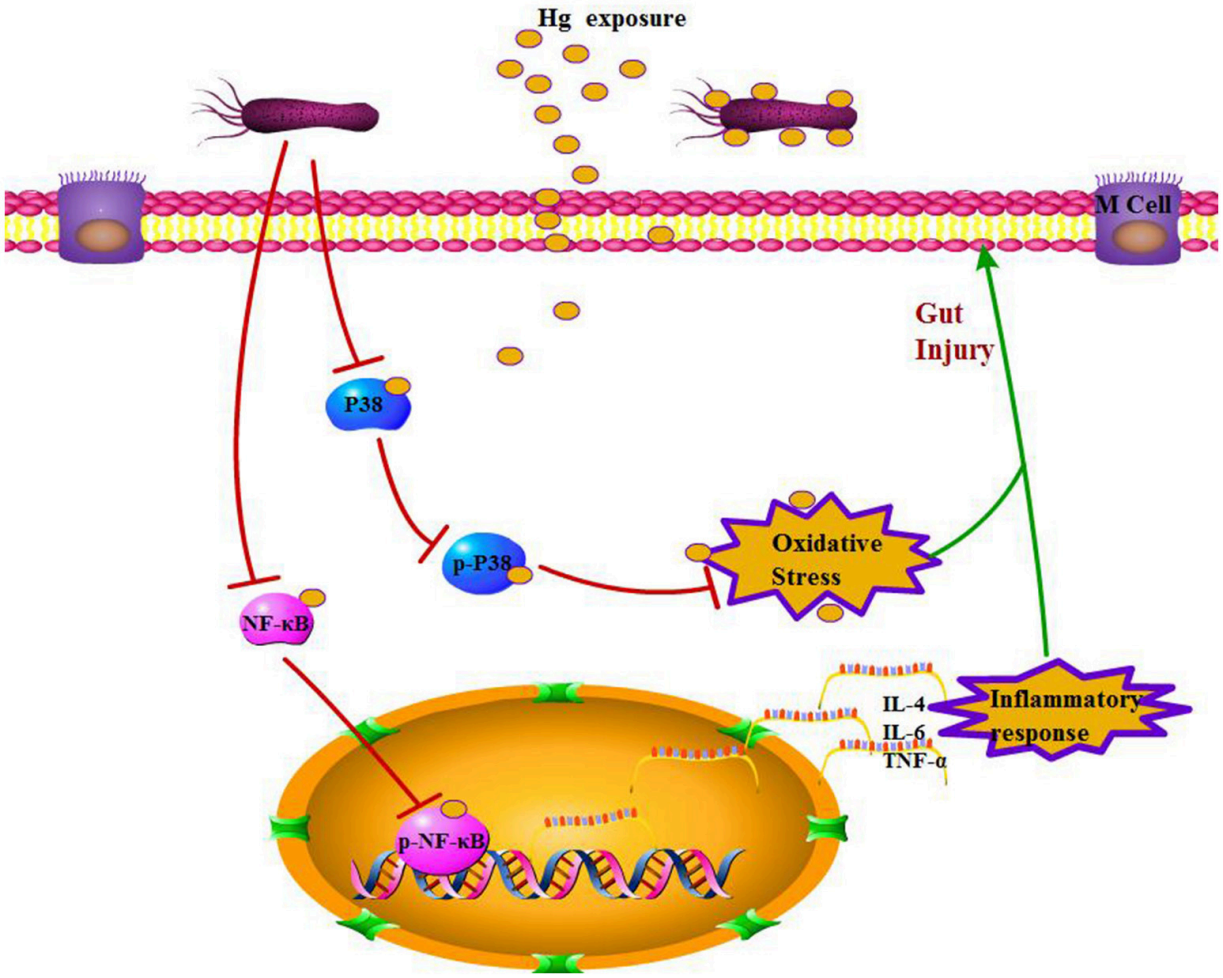
Potential mechanism of *L. brevis* 23017 probiotic effects against Hg-induced gut injury.

There have been many studies on the toxic effects of Hg on different organs, such as the liver, kidneys, muscle, and reproductive system, and toxicity-induced oxidative stress is well documented (Hussain et al., 1999; Aguado et al., 2013; Martinez et al., 2014). Recently, the adverse effects of heavy metal exposure on the intestinal tract have attracted increasing attention because this organ is the first target of heavy metal exposure in the general population. Early studies of heavy metals resulted in histologically evident intestinal toxicity in mice expressed as changes in villi architecture, hemorrhagic gastritis, and intestinal epithelial cell necrosis (Andersen et al., 1988). Similar to other heavy metals, Hg is absorbed by both epithelial cells and *L. brevis* 23017 in the intestines, and an intact gut barrier is crucial to limiting absorption. This study demonstrates the protective effects of *L. brevis* 23017 in response to Hg exposure, which causes significant damage to the gut barrier, such as damaging enterocytes, increasing intestinal permeability, damaging tight junctions, and inducing oxidative stress and inflammatory responses (Figure 6). Previous studies have demonstrated that *Lactobacilli* maintains a normal mucosal barrier via modulation of TJ proteins (Dicksved et al., 2012; Yang et al., 2015). Furthermore, *Lactobacillus* effectively inhibits weight loss in response to Hg exposure. Histopathological examination illustrated that *Lactobacillus* attenuates Hg toxicity compared to the control group with a decrease in mucus thickness, supporting a mechanism of increased gut permeability. Both LAB+Hg-5d and LAB+Hg-12d groups maintained relative integrity in the small intestinal villus of the colon, and the intestinal villus in the LAB+Hg-12d group was much longer than in the LAB+Hg-5d group. Hg exposure significantly decreased expression of occludin and ZO-1 in Hg-5d and Hg-12d groups compared to control groups. Furthermore, various studies of heavy metals have demonstrated that they inhibit the growth of diverse microorganisms (Khan et al., 2010; Fazeli et al., 2011). Moreover, Hg-induced disruption of tight junctions may cause lysosomes to leak from neutrophils, providing an aberrant pathway for expression of inflammatory cytokines. Both inflammatory response and integrity of the intestinal mucosal barrier are closely linked. On the basis of these analyses, *Lactobacillus* protects the gut barrier against Hg toxicity and is important for inhibition of intestinal Hg absorption, also resulting in protection from the inflammatory response and maintenance of intestinal integrity.

There were many studies demonstrating that Hg exposure induces oxidative stress and inflammatory response in different organs (Lund et al., 1993; Huang et al., 2012). Cd causes necrosis or apoptosis of enterocytes, triggering an inflammatory response, disrupting tight junctions in the intestines, and inducing oxidative stress, all of which were reversed by antioxidants (Basuroy et al., 2003; Hyun et al., 2007). In this study, Hg exposure caused oxidative stress by decreasing SOD and GSH activities and promoting higher levels of MDA and IL-10, suggesting that *L. brevis* 23017 promotes an anti-inflammatory phenotype. *L. brevis* 23017 administration directly protects the gut from oxidative stress and inflammatory response, while still significantly inhibiting generation of oxidative stress and pro-inflammatory cytokines, reducing MDA levels and increasing activities of SOD, GSH, and CAT. Oxidative stress also aggravates the inflammatory process (Zhai et al., 2016), and *Lactobacillus plantarum* inhibits absorption of the heavy metal cadmium by preserving the intestinal barrier (Zhai et al., 2014). The research reveals that defense mechanisms against oxidative stress and inflammation are mediated by Nrf2 signaling pathways, which contribute to cellular protection against oxidative stress and reactive metabolites (Fuentes et al., 2015). Collectively, these results show that *L. brevis* 23017 directly protects against Hg-induced oxidative stress and inflammation.

Accumulating evidence supports the theory that *L. brevis* 23017 ameliorates inflammatory bowel disease and that repair of endothelial cells is one of the factors of injury (Feighery et al., 2008; Liu et al., 2010; Greenhill, 2011). Meanwhile, specific metabolites of *Lactobacillus plantarum* suppress pro-inflammatory cytokine production via inhibition of *NF-kappa B* and p38 MAPK expression in murine macrophages (Chon et al., 2010). The present study shows that *L. brevis* 23017 decreases the levels of TNF-α and IL-6, indicating that *L. brevis* 23017 relieves inflammatory responses during Hg exposure in the early phase treatment. Even though there was no significant difference between the LAB+Hg-5d group and LAB+Hg-12d group in terms of IL-6 and TNF-α expression in the duodenum and colon, the IL-6 and TNF-α expression of the LAB+Hg-12d group was lower than the LAB+Hg-5d group. In addition, the increase of IL-10 expression in the LAB+Hg-12d group was significantly higher than the Hg-12d group in the colon. These results showed that the lactobacillus has the anti-inflammation response for a long period. *Lactobacillus curvatus* WiKim38 induces IL-10 production in dendritic cells and alleviates DSS-induced colitis in mice (Jo et al., 2016). And *Lactobacillus fermentum* ZYL0401 could also attenuates lipopolysaccharide-Induced hepatic TNF-α expression and liver injury via an IL-10 mechanism (Jin et al., 2015). There were still some previous studies shown that IL-4 inhibited the TNF-α production (Fujii et al., 2012; Souza et al., 2013).

Additionally, expression of TNF-α, IL-4 and IL-6 were inhibited by *L. brevis* 23017 on *NF-*κ*B* in response to Hg exposure. These findings demonstrate that the *NF-*κ*B* pathway may be involved in the pro-inflammatory effects of Hg exposure in the small intestine. However, increased IL-6 and TNF-α levels induced by Hg were not suppressed by *L. brevis* 23017, suggesting that a unique pathway may exist for Hg to promote the release of IL-6 and TNF-α from the small intestine. Tormos et al., report that p38 MAPK is a sensor of oxidative stress that controls liver regeneration (Tormos et al., 2013). Our results of P38 activation correspond with Hg exposure-induced oxidative stress in the colon, particularly because oral administration of *L. brevis* 23017 blocked the p38 pathway (Hou et al., 2014; Zahran et al., 2017). *L. brevis* 23017 inhibits Hg exposure-induced oxidative stress and triggers MAPK activation in murine gut cells. Importantly, *L. brevis* 23017 reduced the levels of pro-inflammatory cytokines and chemokines induced by Hg exposure via down-regulating *NF-*κ*B* and MAPK activation (Shimazu et al., 2012).

Our results show that Hg selectively activates p38 MAPK and *NF-*κ*B* pathways in different intestinal segments. This finding was supported by our Hg activation of p38 MAPK and *NF-*κ*B* pathways in the 5- and 12-day groups in the colon, but not the duodenum, which were blocked by *L. brevis* 23017 oral administration for 5 days and 12 days. Both p38 MAPK and *NF-*κ*B* pathways were downregulated in response to Hg exposure with concomitant oral administration of *L. brevis* 23017 for 5 days and 12 days in both the colon and duodenum. Oral *L. brevis* 23017 blocked the *NF-*κ*B* pathway much more significantly in the colon compared to the duodenum. *NF-*κ*B* can be activated by different pathways, including p38 MAPK (Ghosh et al., 2009; Olejarz et al., 2014). To confirm the mechanism by which *L. brevis* 23017 regulates *NF-*κ*B* and MAPK pathway activation, we directly measured the effect of *L. brevis* 23017 on phosphorylation of p38 MAPK. Our results showed that Hg exposure promoted activation both of P38 MAPK, *NF-*κ*B* and phosphorylation in the colon but not the duodenum. This trend was the same for P38 MAPK and *NF-*κ*B* pathways. Phosphorylation of p38 MAPK also stimulated phosphorylation of *NF-*κ*B*. This is the first report that Hg exposure activates p38 MAPK and *NF-*κ*B* pathways in different intestinal segments. *L. brevis* 23017 primarily inhibits phosphorylation of mediators such as P38 and *NF-*κ*B* in the duodenum and colon. In particular, Hg exposure is a cumulative toxicity, and *L. brevis* 23017 was superior in relieving both P38 and *NF-*κ*B* pathway activation in the colon.

Most organic mercurial compounds are readily absorbed through lungs and GI tract, and some are readily absorbed through the skin. The intestinal epithelium stem cells, which locate in the intestinal mucosa crypts, have the capacity to self-renew for 2–3 days. The intestinal epithelium cells were not the targeting tissue of mercurial compounds. However, chronic effects of mercury were with the thyroid disruption, liver damage, oxidative stress and lipid metabolism disorder on Bufo gargarizans larvae (Shi et al., 2018). And mercury chronic toxicity might be associated to some cases of hydrocephalus in adult humans. The chronic exposure to mercury takes the oxidative stress, metallothionein induction and organ toxicity in rats (Agrawal et al., 2014). The effects of chronic mercury exposure were on the lysosome system and oxidative stress on the rat kidney (Hendriksen et al., 2007; Akgul et al., 2016).

In conclusion, the present study demonstrates that *L. brevis* 23017 effectively prevents Hg-induced injury by promoting Hg adsorption in the small intestine by reducing intestinal epithelial cell cytotoxicity, maintaining the integrity of TJ proteins, modulating inflammation and alleviating oxidative stress in an animal model. Most importantly, oral Hg administration only activates P38 and *NF-*κ*B* pathways in the colon, with differential results in the intestinal segments, and *L. brevis* 23017 blocks oxidative stress and inflammation through both MAPK and *NF-*κ*B* pathways. These results provide further insight into the mechanisms of inflammation and oxidative stress, offering a potential target for the prevention and treatment of fermented functional foods against oral Hg exposure to protect gut health in daily life.

## Author contributions

XJ and JG designed the research. XH and DL analyzed the data. LZ, SG, and SX performed the research. XJ and JG wrote the paper. XH and HC contributed new reagents and analytic tools.

### Conflict of interest statement

The authors declare that the research was conducted in the absence of any commercial or financial relationships that could be construed as a potential conflict of interest.
